# Obituary

**Published:** 1878-07

**Authors:** 


### OBITUARY.

Dr. J. J. Menefee was born in the State of Indiana; he practiced dentistry twenty-two years, and has lived and practiced his profession for the last fourteen years in San Jose, Cal. He died Oct., 27, 1877, deeply regretted and much beloved as a genial gentleman, a skillful practitioner, a kind husband and loving father.

He was a useful member of the Cal. State Dental Association, where he labored and held official position more or less since its formation.

